# Ability of patients with acute ischemic stroke to recall given information on intravenous thrombolysis: Results of a prospective multicenter study

**DOI:** 10.1177/23969873221143856

**Published:** 2023-01-06

**Authors:** Luzie Schuster, Fabian Essig, Naeimeh Daneshkhah, Juliane Herm, Simon Hellwig, Matthias Endres, Ulrich Dirnagl, Frank Hoffmann, Dominik Michalski, Waltraud Pfeilschifter, Christian Urbanek, Gabor C Petzold, Timolaos Rizos, Andrea Kraft, Karl Georg Haeusler

**Affiliations:** 1 Department of Neurology, University of Leipzig, Leipzig, Germany; 2 Department of Neurology, Universitätsklinikum Würzburg, Würzburg, Germany; 3 Klinik und Hochschulambulanz für Neurologie, Charité - Universitätsmedizin Berlin, Berlin, Germany; 4 Center for Stroke Research Berlin, Charité - Universitätsmedizin Berlin, Berlin, Germany; 5 German Center for Cardiovascular Diseases (DZHK), Partner Site Berlin, Germany; 6 Berlin Institute of Health at Charité - Universitätsmedizin Berlin, Berlin, Germany; 7 German Center for Neurodegenerative Diseases (DZNE), Partner Site Berlin, Germany; 8 QUEST Center, Berlin Institute of Health (BIH), Berlin, Germany; 9 Department of Neurology, Martha-Maria Hospital, Halle/Saale, Germany; 10 Department of Neurology, University of Frankfurt, Frankfurt/Main, Germany; 11 Department of Neurology, Hospital Lüneburg, Lüneburg, Germany; 12 Department of Neurology, Hospital Ludwigshafen, Ludwigshafen am Rhein, Germany; 13 Division of Vascular Neurology, Department of Neurology, University of Bonn, Bonn, Germany; 14 German Center for Neurodegenerative Diseases (DZNE), Bonn, Germany; 15 Department of Neurology, University of Heidelberg, Heidelberg, Germany

**Keywords:** Ischemic stroke, alteplase, informed consent, intracranial bleeding, rt-PA

## Abstract

**Introduction:**

Intravenous thrombolysis (IVT) is an on label treatment for selected patients with acute ischemic stroke (AIS). As major bleeding or allergic shock may occur, the need to ensure patients’ informed consent for IVT is a matter of debate.

**Patients and methods:**

Prospective investigator-initiated multi-center observational study to assess the ability of AIS patients to recall information, provided by a physician during a standardized educational talk (SET) on IVT use. The recall of 20 pre-defined items was assessed in AIS after 60–90 min (*n* = 93) or 23–25 h (*n* = 40) after SET. About 40 patients with subacute stroke, 40 non-stroke patients, and 23 relatives of AIS patients served as controls, and were surveyed 60–90 min after SET.

**Results:**

Within 60–90 min after SET, AIS patients (median age 70 years, 31% female, median NIHSS score on admission 3 points) who were considered capable to provide informed consent recalled 55% (IQR 40%–66.7%) of the provided SET items. In multivariable linear regression analysis recapitulation by AIS patients was associated with their educational level (β = 6.497, *p* < 0.001), self-reported excitement level (β = 1.879, *p* = 0.011) and NIHSS score on admission (β = −1.186, *p* = 0.001). Patients with subacute stroke (70 years, 40% female, median NIHSS = 2) recalled 70% (IQR 55.7%–83.6%), non-stroke patients (75 years, 40% female) 70% (IQR 60%–78.7%), and AIS relatives (58 years, 83% female) 70% (IQR 60%–85%). Compared to subacute stroke patients, AIS patients less often recalled the frequency of IVT-related bleeding (21% vs 43%), allergic shock (15% vs 39%), and bleeding-related morbidity and mortality (44% vs 78%). AIS patients recalled 50% (IQR 42.3%–67.5%) of the provided items 23–25 h after SET.

**Conclusion:**

AIS patients eligible for IVT remember about half of all SET-items after 60–90 min or 23–25 h, respectively. The fact that the recapitulation of IVT-associated risks is particularly poor should be given special consideration.

## Introduction

Intravenous thrombolysis (IVT) using alteplase is the standard therapy for selected patients with acute ischemic stroke (AIS) within a 4.5-h time-window and about 200,000 stroke patients per year in Europe and the United States receive alteplase.^1–3^ Randomized controlled trials have shown that IVT improves the functional outcome in a time-dependent manner, being more effective the earlier treatment is started.^4^ IVT may have severe, sometimes life-threatening side-effects, and the preference for IVT increases with increasing stroke severity, previous stroke, advanced age, and is higher in persons who live with a partner.^5^ As not all stroke patients are able to consent to IVT, the treating physicians are forced to evaluate the presumed will of the patient and to obtain patients’ informed consent.^6–8^ According to German law (BGB §630e), the Mental Capacity Act 2005 in UK and the principle of informed consent in the United States,^9^ the treating physician is obliged to inform the patient on type, scope, implementation, expected consequences, and risks of the medical measure as well as its necessity, urgency, suitability, and prospects of success, which regularly prolongs the door-to-needle time.^10^ Also in line with German law, the US emergency doctrine of implied consent and the guidelines of the American Medical Association/American Stroke Association, the treating physician is not obliged to obtain the patient’s consent in emergency situations before a medical intervention, if the intervention cannot be postponed.^11,12^ Nevertheless, diverging practice to ensure informed consent is obvious, and there is no consensus about the need of informed consent for IVT use.^8,13–15^ These observations were confirmed by a recent survey including 111 designated US stroke centers.^16^ Of note, time-window after onset of stroke-related symptoms and even bed capacities of the treating hospital were found to impact on the physicians’ belief that an informed consent procedure is needed for IVT.^16^

Generally, the ability of a patient to provide consent requires the capacity and willingness to absorb new information, the ability to adequately process provided information and to memorize it even in the context of a potentially life-threatening situation.^6^ However, there are numerous reports demonstrating difficulties of information transfer to patients in the context of acute or even life-threatening illness,^17–19^ as psychological and physical stress as well as the level of education significantly influences the ability of patients to understand provided information.^20,21^ Furthermore, pre-existing or stroke-related acute neurological deficits, such as cognitive decline, speech disorders, reduced level of consciousness as well as disturbed vision, are relevant to the informed consent process.^15,22^ In addition, the ability of patients to provide informed consent depends on the quality and quantity of the informed consent procedure.^23^

Although shared decision-making has gained attention in the field of stroke,^7^ no standardized method exists to assess the understanding of a standardized educational talk (SET) regarding IVT use for AIS.^10,13,16^ As it is not systematically assessed how much information can be absorbed and repeated by a patient in the acute phase of stroke, we conducted a multi-center observational study, and prospectively analyzed the extent to which conveyed information on IVT use during a structured SET can be recapitulated by a patient or a relative of a stroke patient.

## Methods

### Ethical conduct and design of the study

The investigator-initiated, prospective “*Information Recall on Informed Consent to Intravenous Thrombolysis in Patients With Acute Ischaemic Stroke*” observational study (NCT03246256) was approved by the ethics committees of all participating sites, and first by the Charité Ethics Committee (EA4-140-16), Berlin, Germany. All study participants and relatives of participating stroke patients had a sufficient knowledge of German language and gave written informed consent.

**Figure 1.**
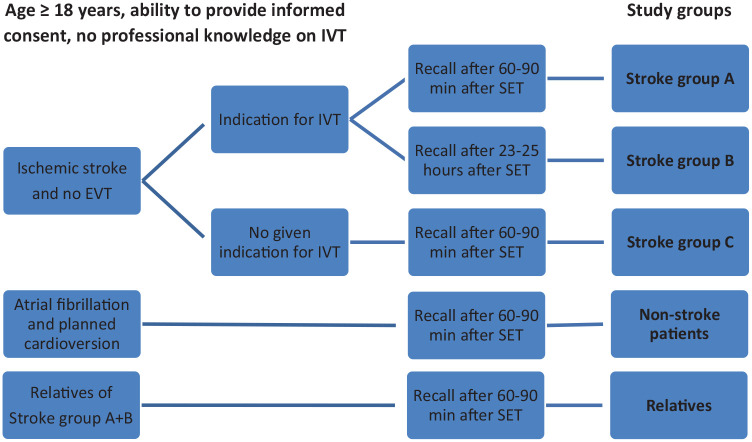
Study groups according to inclusion and exclusion criteria and timing of recall. EVT: endovascular treatment; IVT: intravenous thrombolysis.

Men or women ⩾18 years of age were eligible for study enrollment if they had an ischemic stroke^24^ with given indication for IVT within 4.5 h of symptom onset, did not receive endovascular treatment, were admitted to one of the participating nine German stroke centers and had a sufficient knowledge of German language. AIS patients gave written informed consent within 60–90 min (stroke group A) and within 23–25 h (stroke group B) after SET, respectively. Depending on timing of recall of SET-items participants were divided into different groups (Figure 1). Patients included in stroke group A were asked to recall SET-items within 60–90 min after SET. Patients included in stroke group B were asked to recall SET-items within 23–25 h after SET. Participating centers were advised to enroll patients to study groups A and B. The allocation was based exclusively on the feasibility of interviewing the patients in the given time frame of either 60–90 min or 23–25 h after the SET, respectively. Patients were eligible for stroke group C, if they had an ischemic stroke but no option to undergo IVT. SET was performed 49 h (IQR 22–74 h) after hospital admission. Patients were eligible for non-stroke group D, if they were aged 18 years and older and were admitted to the Department of Cardiology at the Charité, Campus Benjamin Franklin, Berlin, Germany. SET was performed 55 h (IQR 27–101 h) after hospital admission. Volunteers were eligible for enrollment, if they were aged ⩾18 years and a first- or second-degree relative of an AIS patient enrolled into stroke group A or B. Patients or relatives were not available for inclusion if they had undergone IVT before the index stroke or had professional knowledge about IVT. Patients in stroke group C, non-stroke patients as well as relatives were asked to recall SET-items within 60–90 min after standardized SET on IVT use by a trained study physician.

### Standardized educational talk (SET) on IVT

Study centers were instructed to inform treating physicians about the study and to name all pre-defined items during the SET. The provided information consisted of seven categories: the treating physician (name and position), stroke diagnosis using brain imaging (impaired blood circulation, emergency situation), IVT contraindications (anticoagulation, recent stroke or operation, symptom onset >4.5 h), on-label intravenous application of IVT to reopen vessel occlusion, potential IVT benefits (number needed to treat, improvement possible not certain), IVT risks (major bleeding, allergic shock, death) and the patients’ option to reject IVT treatment. Further details can be found in Supplemental Table 1.

### Data assessment

Collected information included patients’ demographics, stroke severity (assessed by the National Institutes of Health Stroke Scale (NIHSS) score^25^ on admission, at the time of recall after SET, and at hospital discharge) and educational level. Collected information of relatives included age, gender, degree of relationship, and educational level. Additionally, the number of queries by the patient/relative during SET and the subjective excitement level during SET was documented in all groups (using a non-validated scale of 0 (no excitement) to 10 (maximal excitement)).

### Hypotheses and primary outcome measure

The following hypotheses were predefined: Primary hypothesis: In AIS patients capable to provide informed consent and without previous thrombolysis, 50% of the mentioned facts during an informed consent interview for IVT can be recalled by the patient within 60–90 min. Secondary hypotheses: (I) In first- or second-degree relatives of stroke patients capable to provide informed consent and without previous stroke, 75% of the mentioned facts during an informed consent interview for IVT can be recalled. (II) In patients capable to provide informed consent with subacute ischemic stroke without previous thrombolysis, 75% of the mentioned facts during an informed consent interview for IVT can be recalled. (III) In AIS patients capable to provide informed consent and without previous thrombolysis, 50% of the mentioned facts during an informed consent interview for IVT can be recalled by the patient within 23–25 h after SET. Primary outcome measure: Recall of the designated facts given during informed consent procedure on IVT after acute ischemic stroke (time frame: within 24 h). The study protocol can be found (in German) in the Supplement.

### Statistical methods and sample size

Based on the hypothesis that stroke patients can reproduce 50% of the facts conveyed during SET and that the standard deviation for factual knowledge in the patient group is 30%, a 95% confidence interval for the mean percentage factual knowledge can be determined with an accuracy of ±6% (44%–56%) with 100 patients. For the secondary hypotheses and other groups, mean values for recalled factual knowledge from SET and 95% confidence interval were calculated. Therefore, we aimed for 100 patients in stroke group A (primary outcome measure), 40 patients in stroke group B, stroke group C, and non-stroke group D. Furthermore, we intended to enroll 40 relatives of patients enrolled into stroke group A or B. Statistical analysis was performed with Graph Pad Prism 8.0.2 (Graph Pad Software, San Diego, CA, USA). Normal distribution of the datasets was tested using the Kolmogorov-Smirnov normality test for significance. Kruskal-Wallis test with correction for multiple comparisons was performed to test for significance. Multivariable linear regression analysis of factors independently associated with the percentage of correctly remembered items were performed using SPSS Statistics. Educational level, NIHSS on admission, age, self-reported excitement level, SET duration (stroke group A, B, C) and educational level, age, self-reported excitement level, SET duration (Non-stroke D, relatives) were used as independent variables, backward selection was performed. Two-sided *p*-values <0.05 were considered statistically significant.

## Results

### Study cohort

Of 180 stroke patients enrolled in group A–C, six patients were excluded due to violation of standardized information transfer, and one patient withdraw informed consent. Baseline demographics of the analyzed cohort including 173 stroke patients, 40 non-stroke patients, and 23 relatives are presented in Table 1. Relatives included children (*n* = 13), grand-children (*n* = 6), or parents (*n* = 2) of AIS patients, as well as one sibling and one spouse.

**Table 1.** Baseline characteristics of enrolled patients and relatives of stroke patients.

	Stroke patients group A–C (*n* = 173)	Stroke patients group A (*n* = 93)	Stroke patients group B (*n* = 40)	Stroke patients group C (*n* = 40)	Non-stroke patients group D (*n* = 40)	Relatives of stroke patients (*n* = 23)
Age, years, median (IQR)	68 (59–78)	70 (63–79)	65 (57–76)	70 (58–77)	75 (72–80)	58 (50–66)
Female sex, *n* (%)	59 (34)	29 (31)	14 (35)	16 (40)	16 (40)	19 (83)
NIHSS score on admission, points, median (IQR)	2 (1–4)	3 (2–5)	3 (2–6)	2 (0–4)	Not applicable	Not applicable
NIHSS score during SET, points, median (IQR)	2 (0–3)	2 (1–4)	1 (0–2)	0 (0–2)	Not applicable	Not applicable
Intravenous thrombolysis, *n* (%)	132 (76)	93 (100)	39 (98)	0 (0)	0 (0)	Not applicable
Education, *n* (%)						
Eighth class	42 (24)	28 (30)	7 (18)	7 (18)	9 (23)	6 (26)
Tenth class	56 (32)	33 (35)	13 (33)	10 (25)	15 (38)	6 (26)
High-school diploma	27 (16)	10 (11)	10 (25)	7 (18)	5 (13)	2 (9)
Academic degree	34 (20)	18 (19)	4 (10)	12 (30)	11 (28)	2 (9)
No degree	7 (4)	1 (1)	4 (10)	2 (5)	–	5 (22)
Not specified	7 (4)	3 (3)	2 (5)	2 (5)	–	2 (9)
Named items, *n*, median (IQR)	20 (16–20)	20 (16–20)	17 (15–19)	20 (20–20)	20 (19–20)	20 (18–20)
SET duration, min, median (IQR)	3 (2–5)	3 (2–4)	3 (2–6)	5 (3–7)	6 (5–7)	5 (4–9)
Number of queries by the patient/relative, *n*, median (IQR)	0 (0–0)	0 (0–0)	0 (0–0)	0 (0–1)	1 (1–2)	0 (0–0)
Subjective excitement level during SET, median (IQR)^a^	2 (0–5)	1 (0–4)	4 (2–5.75)	0 (0–2)	0 (0–2)	4 (3–7.5)

a Patients were asked to rate their excitement level during SET on a scale of 0 (none) to 10 (very excited).

Median age of stroke patients (68 years (IQR 59–78) in groups A–C) was lower compared to non-stroke patients (75 years (IQR 71–80), *p* = 0.03), while relatives of stroke patients were younger (58 years (IQR 48–66), *p* = 0.004) and more often female (17% vs 66%, *p* < 0.001) than stroke patients. The median NIHSS score on admission was higher in 93 stroke group A patients (3 points (IQR 2–5), *p* = 0.01) and 40 stroke group B patients (3 points (IQR 2–6), *p* = 0.04) than in 40 stroke group C (2 points (IQR 0–4)) but not different in stroke group A versus B (*p* = 0.99). The median NIHSS scale score at the time of recalling physician-provided information was higher in stroke group A patients (2 points (IQR 1–4) than in stroke group B patients (1 point (IQR 0–2), *p* = 0.005) or in stroke group C (0 points (IQR 0–2), *p* < 0.001) but not different in stroke group B vs C (*p* = 0.37)). Education level was not different between stroke patients and non-stroke patients (*p* = 0.99), or relatives (*p* = 0.19), respectively.

### SET regarding IVT

SET duration was significantly shorter in AIS (stroke group *A* + *B*, 3 min (IQR 2–5)) versus patients with subacute stroke (stroke group C, 5 min (IQR 3–7), *p* < 0.001), versus non-stroke patients (6 min (IQR 5–7), *p* < 0.001) or versus relatives (5 min (IQR 3–9; *p* = 0.002)). SET duration was not different for stroke group A versus B (*p* = 0.98) (Table 1). The median number of 20 pre-defined items (see Table 3 for details) named during the SET regarding IVT was not different in stroke patients versus non-stroke patients (*p* = 0.12) or relatives (*p* = 0.99) (Table 1). The number of queries was lower in stroke group *A* + *B*, 0 (IQR 0–0) versus stroke group C 0, (IQR 0–2, *p* < 0.001) or non-stroke patients, 1 (IQR 1–2, *p* < 0.001). No difference was found for stroke group *A* + *B* versus relatives (*p* = 0.99), and stroke group A versus B (*p* = 0.99).

### Ability of stroke patients to recall information on IVT

**Figure 2.**
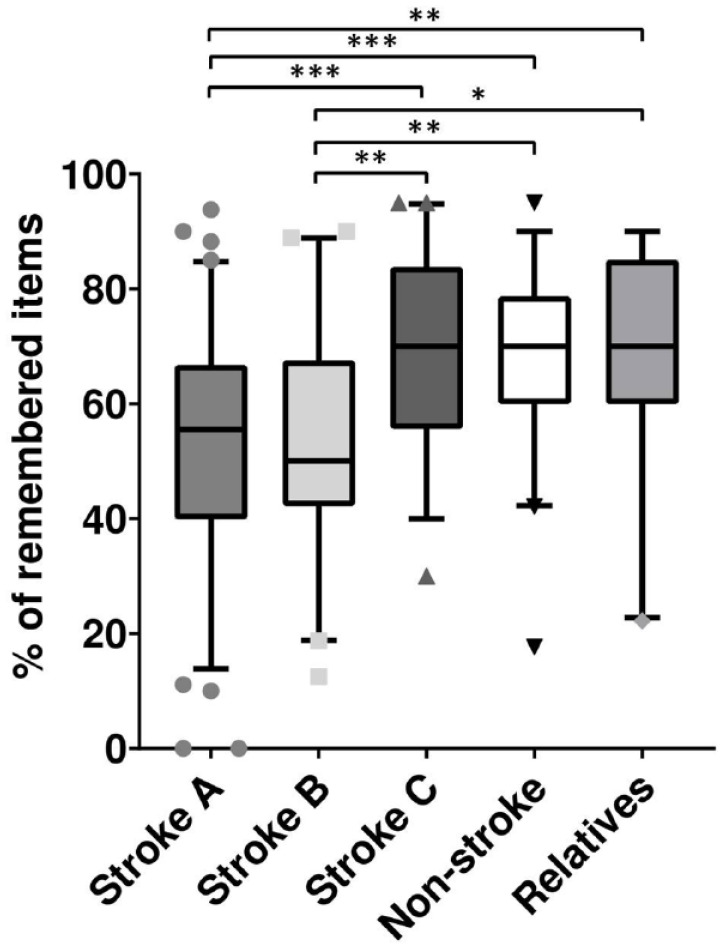
Percentage of recalled information on IVT in the respective study groups. Within boxplots, horizontal lines represent the median, boxes represent values within the 25th–75th percentiles and error bars indicate the range within 5th–95th percentile. Test for significance was performed by Kruskal-Wallis test with correction for multiple comparisons, *p < 0.05, **p < 0.01, ***p < 0.001.

Patients in stroke group A recalled 55% (IQR 40%–66.7%) of all mentioned items within 60–90 min after SET (Figure 2). In a multivariable linear regression analysis (*r*^2^ = 0.199) using backward selection (including educational level, NIHSS score on admission, age, self-reported excitement level, and SET duration), the percentage of remembered SET items was associated with educational level (β = 6.497, *p* < 0.001), self-reported excitement level (β = 1.879, *p* = 0.011), and the NIHSS score on admission (β = −1.186, *p* = 0.001) (Table 2).

**Table 2.** Multivariable linear regression analysis of factors independently associated with the percentage of correctly remembered items.

Independent variable	Coefficient (95% CI)	*p* Value
Stroke patients group A–C		
Age	−0.365 (−0.589 to −0.140)	0.002
Educational level	4.404 (1.922 to 6.886)	0.001
NIHSS score on admission (per point)	−1.186 (−2.041 to 0.332)	0.007
Stroke patients group A		
Educational level	6.497 (2.987 to 10.007)	<0.001
NIHSS score on admission (per point)	−1.050 (−2.148 to 0.048)	0.061
Subjective excitement level	1.879 (0.450 to 3.307)	0.011
Stroke patients group B		
NIHSS score on admission (per point)	−2.154 (−4.072 to 0.237)	0.029
Stroke patients group C		
Age	−0.372 (−0.672 to −0.071)	0.017
SET duration	−1.649 (−2.980 to −0.318)	0.017
Non-stroke patients group D		
Age	−0.493 (−0.901 to −0.086)	0.019
Subjective excitement level	−2.982 (−5.520 to −0.445)	0.022

*R*^2^ = 0.19 for model stroke patients group A–C, *R*^2^ = 0.2 for model stroke patients group A, *R*^2^ = 0.14 for model stroke patients group B, *R*^2^ = 0.31 for model stroke patients group C, *R*^2^ = 0.24 for model non-stroke patients group D.

Patients in stroke group B recalled 50% (IQR 42.3%–67.5%) of all mentioned items within 23–25 h after SET. In multivariable linear regression analysis (*r*^2^ = 0.137), the percentage of remembered SET items was associated with the NIHSS score on admission (β = −2.154, *p* = 0.029). Patients in stroke group C recalled 70% (IQR 55.7%–83.75%) of all mentioned items within 60–90 min after SET. The percentage of remembered items was associated (*r*^2^ = 0.306) with patients’ age (β = −0.372, *p* = 0.017) and SET duration (β = −1.649, *p* = 0.017) (Table 2).

**Figure 3.**
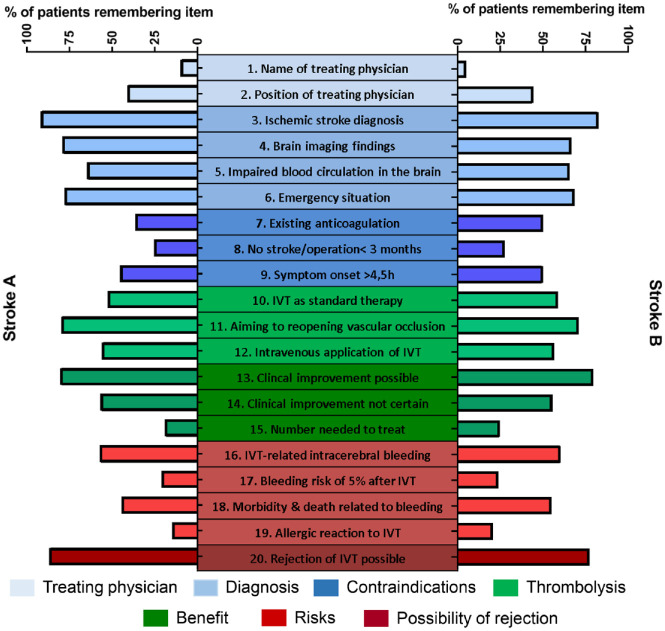
Ability of patients with acute ischemic stroke to recall given information on IVT before the start of IVT at 60–90 min (left, stroke group A) or 23–25 h (right, stroke group B) after SET, respectively. Data is given in % of patients remembering the respective item.

Comparison of stroke group A to stroke group B (Figure 2) revealed no significant differences regarding the percentage of remembered items (55% (IQR 40%–66.7%) in A vs 50% (IQR 42.3%–67.5%) in B, *p* = 0.99). The recall of categorized items (Table 3) or the 20 named items itself (Figure 3) was similar 60–90 min or 23–25 h after SET. The name of the treating physician (10% in stroke group A vs 5% in stroke group B), history of pre-existing stroke or operations within 3 months (25% in A vs 28% in B), the number needed to treat (19% in A vs 25% in B), frequency of bleeding risk (21% in A vs 24% in B), or risk of allergic shock (15% in A vs 21% in B) were recalled by the minority of AIS patients.

**Table 3.** Ability of patients with acute ischemic stroke (stroke group A: *n* = 93, stroke group B: *n* = 40), patients with subacute ischemic stroke (stroke group C: *n* = 40), non-stroke patients (*n* = 40), and relatives of stroke group A or B (*n* = 23) to recall given information on IVT at 60–90 min or 23–25 h (stroke group B only).

Patients remembering named items	Stroke group A	Stroke group B	Stroke group C	Non-stroke group D	Relatives
Treating physician, %	**∑25**	**∑24**	**∑56**	**∑63**	**∑50**
Name of treating physician, *n* (%)	9/91 (10)	2/38 (5)	16/40 (40)	21/40 (53)	8/22 (36)
Position of treating physician, *n* (%)	34/83 (41)	16/36 (44)	29/40 (73)	29/40 (73)	14/22 (64)
Diagnosis, %	**∑78**	**∑71**	**∑89**	**∑81**	**∑88**
Ischemic stroke diagnosis, *n* (%)	85/93 (91)	33/40 (83)	39/40 (98)	38/40 (95)	23/23 (100)
Brain imaging findings, *n* (%)	71/90 (79)	24/36 (67)	37/40 (93)	19/38 (50)	20/22 (87)
Impaired blood circulation, *n* (%)	55/86 (64)	23/35 (66)	35/40 (88)	38/40 (95)	17/22 (77)
Emergency situation, *n* (%)	66/85 (78)	26/38 (68)	31/39 (79)	33/40 (83)	19/22 (86)
Contraindications, %	**∑35**	**∑42**	**∑60**	**∑60**	**∑55**
No existing anticoagulation, *n* (%)	30/83 (36)	16/32 (50)	24/39 (62)	30/39 (77)	11/21 (52)
No stroke/operation <3 month, *n* (%)	21/83 (45)	10/36 (28)	18/40 (45)	15/38 (40)	7/20 (35)
Symptom onset >4.5 h, *n* (%)	32/71 (45)	15/30 (50)	29/40 (73)	25/40 (63)	16/21 (76)
Thrombolysis, %	**∑63**	**∑63**	**∑68**	**∑71**	**∑80**
IVT as standard therapy, *n* (%)	44/84 (52)	20/34 (59)	22/40 (55)	22/40 (55)	17/21 (81)
Aim to reopen vascular occlusion, *n* (%)	70/88 (80)	27/38 (71)	32/40 (80)	34/40 (85)	20/22 (91)
Intravenous application of IVT, *n* (%)	49/88 (56)	17/30 (57)	27/39 (69)	27/37 (73)	13/19 (68)
Benefit, %	**∑56**	**∑62**	**∑73**	**∑78**	**∑71**
Clinical improvement possible, *n* (%)	72/90 (80)	31/39 (80)	34/40 (85)	39/40 (98)	20/22 (91)
Clinical improvement not certain, *n* (%)	43/76 (57)	20/36 (56)	34/40 (85)	34/40 (85)	17/20 (85)
Number needed to treat, *n* (%)	11/58 (19)	3/12 (25)	20/40 (50)	14/32 (44)	5/17 (29)
Risks, %	**∑35**	**∑43**	**∑57**	**∑48**	**∑55**
IVT-related intracerebral bleeding, *n* (%)	52/91 (57)	23/38 (60)	27/40 (68)	28/40 (70)	17/21 (81)
Bleeding risk of 5% after IVT use, *n* (%)	15/72 (21)	6/25 (24)	17/40 (43)	11/39 (28)	7/20 (35)
Bleeding-related morbidity and death, *n* (%)	31/70 (44)	16/29 (55)	31/40 (78)	32/38 (84))	11/18 (61)
Allergic shock to IVT, *n* (%)	11/82 (14)	5/24 (21)	15/39 (39)	4/38 (11)	7/18 (39)
Rejection of IVT use possible, %	**∑87**	**∑77**	**∑93**	**∑98**	**∑90**
Rejection of IVT use possible, *n* (%)	71/82 (87)	27/35 (77)	37/40 (93)	39/40 (98)	17/19 (90)

Given *n* of each item refers to correctly remembered items in all patients/relatives. The percentage of the correctly remembered item is shown in brackets. The percentage of correctly remembered item per topic is shown in bold letters.

Comparison of stroke group A to stroke group C revealed significant differences regarding the percentage of remembered items 60–90 min after SET (55% (IQR 40–66.7%) in A vs 70% (IQR 55.7–83.75%) in C, *p* < 0.001; Figure 2). Categorizing provided information, numerical differences were obvious regarding the treating physician (25% in A vs. 56% in C), IVT contraindications (35% in A vs. 60% in C) and IVT-related risks (35% in A vs. 57% in C; Table 3, Supplemental Figures 1 and 2). In detail, the frequency of IVT-related bleeding (21% in A vs. 43% in C), allergic risk (15% in A vs. 39% in C) or bleeding-related morbidity and mortality (44% in A vs. 78% in C) was less often recalled.

### Ability of non-stroke patients or relatives to recall information on IVT

Non-stroke patients recalled 70% (IQR 60%–78.7%) of all mentioned items within 60–90 min after SET, while relatives recalled 70% (IQR 60%–85%; Table 3, Supplemental Figures 1 and 2). For non-stroke patients, the multivariable linear regression analysis (*r*^2^ = 0.243) using backward selection (including educational level, age, self-reported excitement level, and SET duration), the percentage of remembered SET items was associated with age (β = −0.493, *p* = 0.019) and subjective excitement level (β = −2.982, *p* = 0.022; Table 2). Multivariable linear regression analysis for relatives revealed no significance (data not shown).

### Primary and secondary hypotheses

The primary study hypothesis that AIS patients are capable to recall 50% of all SET items was met in stroke group A as well as in stroke group B. The secondary study hypotheses that patients with subacute ischemic stroke or without stroke are capable to recall 75% of all SET-items was rejected in stroke group C, non-stroke patients or relatives.

### Ability to recall information on IVT across groups

Comparing stroke group A to non-stroke patients (55% (IQR 40%–66.7%) in A vs. 70% (IQR 60%–78.7%) in D, *p* < 0.001) and relatives (55% (IQR 40%–66.7%) in A vs. 70% (IQR 60%–85%), *p* = 0.003) revealed significant differences regarding the percentage of remembered items. The recall of categorized items numerically differed regarding the treating physician (24% in stroke group A vs. 63% in non-stroke patient group D vs. 50% in relatives), IVT contraindications (35% in A vs. 60% in D vs. 59% in relatives), and IVT benefit (58% in A vs. 78% in D; Table 2).

Comparing stroke group C to non-stroke patients (70% ± 16% in C vs. 68% ± 21% in D, *p* = 0.99) and relatives (70% ± 16% in C vs. 69% ± 20%, *p* = 0.99) revealed no differences regarding the percentage of remembered items. The recall of categories items (Table 3) and the 20 named items itself (Supplemental Figures 1 and 2) were similar.

### Ability to recall SET information regarded to be of “major importance”

Focusing on a subset of 10 SET information regarded to be of “major importance” (Supplemental Table 2), similar results were achieved as reported in the study protocol-based approach (Supplemental Table 3 and Supplemental Figure 2). Within 60–90 min after SET, AIS patients recalled 60% (IQR 44%–76%) of the provided SET items regarded to be of major importance. In multivariable linear regression analysis recapitulation of these items by AIS patients was associated with their educational level (β = 5.130, *p* = 0.015), and self-reported excitement level (β = 1.780, *p* = 0.031) but not with the NIHSS score on admission (β = −0.797, *p* = 0.2) or age (β = −0.35, *p* = 0.082). Patients with subacute stroke recalled 75% (IQR 43%–80%), non-stroke patients 70% (IQR 60%–80%), and AIS relatives 80% (IQR 67%–100%). AIS patients recalled 56% (IQR 43%–80%) of the provided items regarded to be of major importance 23–25 h after SET.

## Discussion

The need to ensure patients’ informed consent for IVT is a matter of debate.^10,13,15,16,22,26,27^ This prospective multi-center observational study demonstrates that IVT-eligible patients with mild to moderate ischemic stroke are able to recall about half of the information provided by a trained physician during a SET within 4.5 h after stroke onset.

The extent of recalled information in AIS patients was similar after 60–90 min or 23–25 h after SET, respectively. After 60–90 min, fewer items were recalled by patients with lower educational level, higher NIHSS score on admission and patients with lower self-reported excitement level. After 23–25 h, fewer items were recalled by patients with higher NIHSS score on admission. Clustering SET-information in seven categories revealed that acute stroke patients less often remembered given information on the treating physician, IVT contraindications and IVT-related risks, like major bleeding frequency or allergic shock. Clustering SET-information with regard to subjective clinical importance (focusing on potential harms and benefits of IVT use) revealed similar results to the protocol-based approach.

If compared to patients with AIS, patients with subacute stroke, hospitalized patients without stroke or first- or second-degree relatives of enrolled AIS patients recalled more information at 60–90 min after SET. Interestingly, categorized items were recalled in a similar fashion in these cohorts. In patients with subacute stroke or hospitalized patients without stroke, fewer items were recalled by elderly patients.

Whether or not improvement of information transfer during SET is possible by using graphical illustrations has to be tested in future trials.^28–31^ At present, the question remains whether or not the information needed to provide informed consent on IVT use can be meaningfully communicated to AIS patients.^13,14,17,32,33^ One may argue that our data support the priestly (paternalistic) model of doctor-patient relationship.^34,35^ However, the authors do not believe that the treating physician shall make all medical decisions based on personal expertise. To our mind, consulting the patient (or its legal representative) to assess patient’s interests is of utmost importance, presenting treatment options and sharing the treating physician’s personal recommendation. As demonstrated in the present study, relevant information regarding IVT use in AIS can be provided in a standardized fashion within 3 min in median. This is not in line with a recently published online survey,^10^ reporting a varying content of information transfer lasting more than 5 min in about half of all patients eligible for IVT use in the USA. However, an online survey among Dutch neurologists reported a maximum of 5 min for information transfer.^27^

The following limitations of our prospective multi-center observational study have to be considered: First, the prerequisite for study participation was the presumed ability to understand provided information on IVT as well as the ability to provide written informed consent after 60–90 min or 23–25 h later, respectively. This implies a selection bias toward patients with less severe stroke.^36^ Stroke-related neurological deficits in unselected cohorts may further limit the practicability of knowledge transfer in the hyperacute phase of stroke. Second, On the basis of a follow-up survey we cannot make a reliable statement about the level of information of stroke patients at the time of decision-making for IVT use. Third, seven study participants (of stroke group A) were excluded from the present analysis due to insufficient data transfer during SET. Fourth, four dichotomized questions were used to recall SET-information leading to a presumably higher rate of correct recall. Fifth, SET duration was shorter in AIS patients versus non-AIS cohorts, mainly due to the higher rate of queries in non-stroke patients and patients with subacute stroke. Furthermore, the non-AIS cohort and patients with subacute ischemic stroke (stroke group C) were informed about study enrollment before providing information on IVT use. As study physicians were advised to point out the importance of the upcoming information on IVT to AIS patients (stroke group A and B), we believe that not mentioning the possibility to participate in a clinical study is a non-relevant bias but we are unable to prove this assumption. Sixth, the self-reported excitement level is not based on an established scale. Seventh, as the COVID-19 pandemic excluded almost all relatives from all participating hospitals from early 2020 to mid-2021, the intended recruitment of relatives was not completed as the enrollment of study patients was finalized. Therefore, we cannot draw final conclusions on the group of relatives. However, our data underline the potential importance of relatives of AIS patients in shared decision-making, in addition to the possibility for the attending physician to determine patients’ values and treatment goals.^15^

Finally, the generalizability of the results is limited to a certain extent as all patients and relatives were enrolled in German stroke centers only and women are underrepresented in the present study. Furthermore, patients with wake-up stroke were excluded, as the level of clinical evidence regarding IVT varies to certain extent.^37^

## Conclusion

To the best of our knowledge, this is the first detailed report on the ability of AIS patients to recall information provided during SET on IVT use. As demonstrated, information recall in AIS patients is limited, especially in patients with lower educational level, lower self-reported excitement level and those with higher NIHSS score on admission. The fact that recall of IVT-associated risks is particularly poor should be given special consideration when conducting SET on IVT use. In addition, further efforts are needed to improve information transfer in the context of shared decision making in the hyperacute phase of ischemic stroke in eligible patients for IVT.

## Supplemental Material

sj-docx-1-eso-10.1177_23969873221143856 – Supplemental material for Ability of patients with acute ischemic stroke to recall given information on intravenous thrombolysis: Results of a prospective multicenter study Click here for additional data file. Supplemental material, sj-docx-1-eso-10.1177_23969873221143856 for Ability of patients with acute ischemic stroke to recall given information on intravenous thrombolysis: Results of a prospective multicenter study by Luzie Schuster, Fabian Essig, Naeimeh Daneshkhah, Juliane Herm, Simon Hellwig, Matthias Endres, Ulrich Dirnagl, Frank Hoffmann, Dominik Michalski, Waltraud Pfeilschifter, Christian Urbanek, Gabor C Petzold, Timolaos Rizos, Andrea Kraft and Karl Georg Haeusler in European Stroke Journal

sj-docx-2-eso-10.1177_23969873221143856 – Supplemental material for Ability of patients with acute ischemic stroke to recall given information on intravenous thrombolysis: Results of a prospective multicenter study Click here for additional data file. Supplemental material, sj-docx-2-eso-10.1177_23969873221143856 for Ability of patients with acute ischemic stroke to recall given information on intravenous thrombolysis: Results of a prospective multicenter study by Luzie Schuster, Fabian Essig, Naeimeh Daneshkhah, Juliane Herm, Simon Hellwig, Matthias Endres, Ulrich Dirnagl, Frank Hoffmann, Dominik Michalski, Waltraud Pfeilschifter, Christian Urbanek, Gabor C Petzold, Timolaos Rizos, Andrea Kraft and Karl Georg Haeusler in European Stroke Journal

sj-jpeg-4-eso-10.1177_23969873221143856 – Supplemental material for Ability of patients with acute ischemic stroke to recall given information on intravenous thrombolysis: Results of a prospective multicenter study Click here for additional data file. Supplemental material, sj-jpeg-4-eso-10.1177_23969873221143856 for Ability of patients with acute ischemic stroke to recall given information on intravenous thrombolysis: Results of a prospective multicenter study by Luzie Schuster, Fabian Essig, Naeimeh Daneshkhah, Juliane Herm, Simon Hellwig, Matthias Endres, Ulrich Dirnagl, Frank Hoffmann, Dominik Michalski, Waltraud Pfeilschifter, Christian Urbanek, Gabor C Petzold, Timolaos Rizos, Andrea Kraft and Karl Georg Haeusler in European Stroke Journal

sj-jpg-5-eso-10.1177_23969873221143856 – Supplemental material for Ability of patients with acute ischemic stroke to recall given information on intravenous thrombolysis: Results of a prospective multicenter study Click here for additional data file. Supplemental material, sj-jpg-5-eso-10.1177_23969873221143856 for Ability of patients with acute ischemic stroke to recall given information on intravenous thrombolysis: Results of a prospective multicenter study by Luzie Schuster, Fabian Essig, Naeimeh Daneshkhah, Juliane Herm, Simon Hellwig, Matthias Endres, Ulrich Dirnagl, Frank Hoffmann, Dominik Michalski, Waltraud Pfeilschifter, Christian Urbanek, Gabor C Petzold, Timolaos Rizos, Andrea Kraft and Karl Georg Haeusler in European Stroke Journal

sj-pptx-3-eso-10.1177_23969873221143856 – Supplemental material for Ability of patients with acute ischemic stroke to recall given information on intravenous thrombolysis: Results of a prospective multicenter study Click here for additional data file. Supplemental material, sj-pptx-3-eso-10.1177_23969873221143856 for Ability of patients with acute ischemic stroke to recall given information on intravenous thrombolysis: Results of a prospective multicenter study by Luzie Schuster, Fabian Essig, Naeimeh Daneshkhah, Juliane Herm, Simon Hellwig, Matthias Endres, Ulrich Dirnagl, Frank Hoffmann, Dominik Michalski, Waltraud Pfeilschifter, Christian Urbanek, Gabor C Petzold, Timolaos Rizos, Andrea Kraft and Karl Georg Haeusler in European Stroke Journal
