# Supplementary material for: Ability of patients with acute ischemic stroke to recall given information on intravenous thrombolysis: Results of a prospective multicenter study
Source: Eur Stroke J. 2023 Jan 6;8(1):241–50. doi: 10.1177/23969873221143856 (PMC10069168; doi:10.1177/23969873221143856)
Supplement: sj-docx-1-eso-10.1177_23969873221143856 – Supplemental material for Ability of patients with acute ischemic stroke to recall given information on intravenous thrombolysis: Results of a prospective multicenter study [file sj-docx-1-eso-10.1177_23969873221143856.docx]

**SUPPLEMENT Table 1** Items of the standardized educational talk provided to patients or relatives of stroke patients by a trained physician

| **Nr** | **Category** | **Items of educational talk**  **(named by treating physician)** | **Query of items (by study physicians)** | **Possible answers (by patients or relatives)** |
| --- | --- | --- | --- | --- |
| **1** | Physician | Name of treating physician | How was the name of the treating physician, who gave you medical information about the intravenous thrombolysis? | Name |
| **2** |  | Position of treating physician (resident physician, senior physician, chief physician) | Which position has the physician in hospital, who informed you about the intravenous thrombolysis? | Position |
| **3** | Diagnosis | Ischemic stroke diagnosis | What diagnosis have they been made? | Ischemic Stroke |
| **4** |  | Brain imaging findings | How was the diagnosis of an ischemic stroke made for you? | Computer tomography (CCT) or magnetic resonance imaging (MRI), „Roentgen of the brain“, “picture of the brain“ |
| **5** |  | Impaired blood circulation in the brain | How does an ischemic stroke occur? | Impaired blood circulation, ciruclatory disorder, „dead brain tissue“, no blood supply. |
| **6** |  | Emergency situation | Why was there an urgent need for action or therapy? | Emergency situation, to avoid brain tissue damage/dying, time-dependent increase of damage |
| **7** | Contraindications | Existing anticoagulation | Which drugs make an intravenous thrombolysis impossible and have therefore been asked in advance/during educational talk? | “drugs for blood thinning”, anticoagulant drugs |
| **8** |  | No stroke/operation < 3 months | Which pre-existing conditions make an intravenous thrombolysis impossible and have therefore been asked in advance/during educational talk? | Recent stroke or operation |
| **9** |  | symptom onset >4,5 | For how many hours after symptom onset, intravenous thrombolysis can still be performed? | 4·5 hours |
| **10** | Thrombolysis | Thrombolysis as standard therapy | What is the approved standard therapy for acute ischemic stroke? | thrombolysis, “dissolution of the thrombus“, rt-PA infusion |
| **11** |  | Aiming to reopening vascular occlusion | What is the desired effect of thrombolysis? | “dissolution of the thrombus“, Reopening the vascular occlusion |
| **12** |  | Only Intravenous application of thrombolysis | Would thrombolysis also have been possible by taking an oral drug? | No |
| **13** | Benefit | Clinical improvement possible | May thrombolysis help to reduce stroke-related symptoms? | Yes |
| **14** |  | Clinical improvement not certain | Does thrombolysis lead to an improvement of stroke-associated symptoms in all treated patients? | No |
| **15** |  | Number needed to treat to avoid serious disability – NNT 7-10 | How many patients would have to be treated with thrombolysis to avoid severe disability from ischemic stroke in one patient? | 7-10 |
| **16** | Risks | Thrombolysis related intracerebral bleeding | What is the greatest risk of thrombolysis? | Intracerebral bleeding |
| **17** |  | bleeding risk in 5% of intravenous thrombolysis | How often does intracranial bleeding occur after thrombolysis? | 5%, 1 of 20, 5 of 100 |
| **18** |  | bleeding related morbidity or death possible | What can be the result of an occurred intracerebral bleeding? | Death, Disability, Dependency |
| **19** |  | allergic shock due to intravenous thrombolysis | What other side effects can occur? | allergy, allergic shock |
| **20** | Rejection | rejection of intravenous thrombolysis possible | Have you been informed whether a rejection of the therapy is possible? | Yes |

**SUPPLEMENT Table 2**: Subset of SET information regarded to be of “major importance”.

| **Provided information of major importance** | **Provided information of minor importance** |
| --- | --- |
| Ischemic stroke diagnosis | Name of treating physician |
| Emergency situation | Position of treating physician |
| IVT as standard therapy | Brain imaging findings |
| Clinical improvement possible | Impaired blood circulation in the brain |
| Clinical improvement not certain | No existing anticoagulation |
| IVT-related intracerebral bleeding | No stroke/operation < 3 months |
| Bleeding risk of 5% after IVT use | Symptom onset > 4.5 hours |
| Morbidity & death related to bleeding | Aim to reopen vascular occlusion |
| Allergic shock to IVT | Intravenous application of IVT |
| Refusal of IVT use possible | Number needed to treat |

**SUPPLEMENT Table 3:** Multivariable linear regression analysis of factors independently associated with the percentage of correctly remembered items focusing on the subset of SET information regarded to be of “major importance”.

| Independent variable Coefficient (95%CI) p |
| --- |
| Stroke group A-C  Age -0.428 (-0.680 - -0.176) 0.001  Educational level 3.698 (0.913 - 6.484) 0.01  NIHSS score on admission (per point) -0.869 (-1.828 - 0.090) 0.075 |
| Stroke group A  Age -0.35 (-0.745 - -0.045) 0.082  Educational level 5.130 (1.031 - 9.229) 0.015  Subjective excitement level 1.780 (0.169 - 3.390) 0.031 |
| Stroke group B  NIHSS score on admission (per point) -2.326 (-4.683 - 0.032) 0.053 |
| Stroke group C  Age -0.414 (-0.710 - -0.118) 0.008  SET duration -1.928 (-3.240 - -0.616) 0.005 |
| Non-stroke group D  Age -0.45 (-0.869 - -0.031) 0.036  Subjective excitement level -2.512 (-5.12 - 0.096) 0.059 |

Note: R^2^=0.14 for model stroke patients group A-C, R^2^=0.17 for model stroke patients group A, R^2^=0.11 for model stroke patients group B, R^2^=0.37 for model stroke patients group C, R^2^=0.19 for model non-stroke patients group D
