# Supplementary material for: Ability of patients with acute ischemic stroke to recall given information on intravenous thrombolysis: Results of a prospective multicenter study
Source: Eur Stroke J. 2023 Jan 6;8(1):241–50. doi: 10.1177/23969873221143856 (PMC10069168; doi:10.1177/23969873221143856)
Supplement: sj-docx-2-eso-10.1177_23969873221143856 – Supplemental material for Ability of patients with acute ischemic stroke to recall given information on intravenous thrombolysis: Results of a prospective multicenter study [file sj-docx-2-eso-10.1177_23969873221143856.docx]

Studienprotokoll

| **1. Titel der Studie** |  |
| --- | --- |
| Name der Studie | **Prospektive multizentrische Beobachtungsstudie zur Untersuchung des inhaltlichen Verständnisses eines Aufklärungsgespräches zur Thrombolyse bei**  **ischämischem Schlaganfall (LYSA)** |
| Versionsnummer des Protokolls. | 1.0 vom 28.10.2018 |
| **2. Verantwortlichkeiten** |  |
| …Studienleiter mit Institution  …und Kontaktdaten | Studienleiter:  Univ.-Prof. Dr. med. Karl Georg Häusler Geschäftsführender Oberarzt Neurologische Klinik und Poliklinik Universitätsklinikum Würzburg  Josef-Schneider-Str. 11  97080 Würzburg  Tel. 0931 20123755  [Haeusler_K@ukw.de](mailto:Haeusler_K@ukw.de)  *Adresse bis zum 30.06.2018:*  Centrum für Schlaganfallforschung Berlin (CSB) Oberarzt der Klinik für Neurologie  Charité Campus Benjamin Franklin Tel.: 030 – 450 560 676  Fax: 030 – 450 560 979  [georg.haeusler@charite.de](mailto:georg.haeusler@charite.de) |
| Beteiligte Institutionen | Charité – Universitätsmedizin Berlin (ehemaliges Studienzentrum) Neurologische Klinik und Poliklinik  Campus Mitte Charitéplatz 1  10117 Berlin  Medizinische Klinik II (Kardiologie) Campus Benjamin Franklin Hindenburgdamm 30  12203 Berlin  Klinikum Ludwigshafen Neurologische Klinik Bremserstraße 79  67063 Ludwigshafen am Rhein  Universitätsklinikum Frankfurt Zentrum für Neurologie Schleusenweg 2-16  60528 Frankfurt am Main  Universitätsklinikum Heidelberg Neurologische Klinik  Im Neuenheimer Feld 400 69120 Heidelberg  Zentrum für Neurologie Universitätsklinikum Tübingen Hoppe-Seyler-Str. 3  72076 Tübingen |

|  | Krankenhaus Martha-Maria Halle Dölau Klinik für Neurologie  Röntgenstr. 1  06120 Halle (Saale)  Universitätsklinikum Bonn (AöR) Klinik und Poliklinik für Neurologie Sigmund-Freud-Straße 25  53127 Bonn |
| --- | --- |
| Verantwortliche Mitarbeiter | Studienkoordination :  Frau Dr. med. Luzie Köhler Universitätsklinikum Leipzig  Klinik und Poliklinik für Neurologie Liebigstr. 20  04103 Leipzig  Tel. 0341 9724358  [luzie.koehler@medizin.uni-leipzig.de](mailto:luzie.koehler@medizin.uni-leipzig.de)  Biometrie:  Frau PD Dr. phil. Ulrike Grittner  Institut für Biometrie und Klinische Epidemiologie Charité - Universitätsmedizin Berlin  Campus Mitte Charitéplatz 1  10117 Berlin  Tel: 030 450 56 21 74  [ulrike.grittner@charite.de](mailto:ulrike.grittner@charite.de) |
| **3. Unterschrift des Studienleiters zur Bestätigung des Protokolls** | 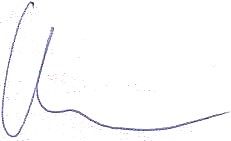  Prof. Dr. med. Karl Georg Häusler |
| **4. Rationale** |  |
| Hintergrund, Stand der Forschung | Die Aufgabe jeden Arztes ist es die Gesundheit seiner Patienten zu schützen. Im Rahmen der ärztlichen Tätigkeit kommt der Aufklärungspflicht gegenüber dem Patienten zum Schutze seiner Selbstbestimmung ein hoher Stellenwert zu. Die Aufklärungspflicht betrifft dabei auch den Einsatz von Medikamenten, deren zeitlich verzögerter Einsatz mit einer verminderten Effizienz einhergehen kann, wie dies für die intravenöse Thrombolyse mit rekombinantem gewebsspezifischem Plasminogenaktivator („Thrombolyse“) der Fall ist. Die Thrombolyse ist laut aktuellen Leitlinien als Standardtherapie bei Patienten mit akutem ischämischem Schlaganfall binnen eines 4,5 Stunden-Zeitfensters anzusehen (Hacke, 2008; Lees, 2010). Allein in Deutschland erhalten pro Jahr etwa 20000 Schlaganfallpatienten diese Therapie. In Studien konnte wiederholt gezeigt werden, dass die Anwendung der Thrombolyse im genannten Zeitfenster das funktionelle Outcome der Patienten verbessern kann (NINDS,1995; Hacke et al. 2004; Hacke 2008). Eine Analyse verschiedener Thrombolyse-Studien (Emberson 2014) konnte zudem belegen, dass die Wahrscheinlichkeit eines guten klinischen Outcomes nach Thrombolyse direkt mit einem schnellen Therapiebeginn korreliert. So ist die Chance auf ein gutes klinisches Outcome nach 3 Monaten bei einer Thrombolyse innerhalb von 3 Stunden nach Symptombeginn etwa 1,75 mal so hoch wie bei Schlaganfallpatienten ohne eine Thrombolyse. Eine Thrombolyse innerhalb von 3-4,5 Stunden nach Symptombeginn erhöht die Wahrscheinlichkeit eines guten klinischen Outcomes hingegen etwa 1,15-fach. Mit der Dauer der Latenz von Symptombeginn des Schlaganfalls bis zur Thrombolyse zeigt sich diese somit als weniger wirksam. Darüber hinaus besteht das Risiko für schwerwiegende Nebenwirkungen, wie beispielsweise eine intrakranielle Blutung, die etwa 4%  aller Schlaganfallpatienten betrifft, die eine Thrombolyse erhalten haben. Diese |

|  | Möglichkeit des Auftretens dieser potentiell lebensbedrohlichen Komplikationen unterstreicht die Bedeutung einer sorgfältigen Aufklärung der betroffenen Patienten. Bei einer ordnungsgemäß durchgeführten Aufklärung mussdaher sichergestellt werden, dass der Patient die Therapie sowie alle damit verbundenen Risiken verstanden hat, um so in der Lage zu sein, eine Entscheidung zu treffen und eigenverantwortlich in die Behandlung einzuwilligen oder diese abzulehnen. Dies stellt die behandelnden Ärzte vor die Herausforderung den betroffenen Patienten in möglichst kurzer Zeit ausführlich und verständlich einen komplexen Sachverhalt nahezubringen.  Eine Einwilligungsfähigkeit basiert zum Einem auf der Fähigkeit und Bereitschaft neue Informationen aufzunehmen und zum anderen auf dem Verstehen und der adäquaten Verarbeitung des im Rahmen der Aufklärung Gehörten bzw. Gelesenen. Eine Einwilligungsfähigkeit macht außerdem die Einordnung der Informationen in den Kontext der potentiell lebensbedrohlichen Akutsituation durch den Patienten unabdingbar (vgl. Akinsanya et al., 2009). Eine Einschätzung der Entscheidungskompetenz des Patienten obliegt dabei den behandelnden Ärzten. Bereits etliche Untersuchungen konnten darlegen, dass in der Patientenaufklärung in Akutsituationen große Schwierigkeiten bestehen (Ciccone, 2003; Knapp et al., 2010; Mamo, 2014) Relevant sind unter anderem ein bestehender Zeitdruck für den Erfolg der Therapie und somit für den Behandler oder auch eine verminderte Aufmerksamkeit des Patienten (Ciccone et al., 2001; Mamo, 2014). Klinische Studien, welche die Einwilligungsfähigkeit bei Patienten mit akutem Herzinfarkt untersucht haben, deuten darauf hin, dass allein psychischer und physischer Stress das inhaltliche Verständnis der betroffenen Patienten maßgeblich beeinflussen (bspw. Agård et al., 2001; Williams et al., 2003). Darüber hinaus konnten auch Cassell et al. (2001) zeigen, dass schwer kranke, hospitalisierte Patienten in Ihrer Fähigkeit zur Entscheidungsfindung deutlich eingeschränkt sind. Eine Übersichtsarbeit, die 34 Studien zu Aufklärung und Einwilligungsfähigkeit im Rahmen von klinischen Interventionen oder Forschungsprojekten untersuchte, postulierte, dass ein besseres inhaltliches Verständnis von der Güte der Aufklärung abhängig ist (Dunn and Jeste, 2001). Bisher existiert kein standardisiertes Verfahren zur Erfassung von Inhalt und Durchführung eines Aufklärungsgespräches zur Thrombolyse nach akutem ischämischem Schlaganfall. Neben dem bestehenden Zeitdruck aufgrund einer inversen Korrelation zwischen Therapiebeginn und des Behandlungserfolges sind Schlaganfall-induzierte Defizite, wie beispielsweise kognitive Störungen  oder Sprachstörungen von hoher Relevanz. |
| --- | --- |
| … Begründung für die Studie | Aktuell ist unklar, in welchem Ausmaß Informationen durch Schlaganfallpatienten in der Akutsituation wirklich aufgenommen und verarbeitet werden können. Die geplante prospektive Datenerhebung soll im Vergleich der verschiedenen Zielgruppen diesbezügliche Informationen liefern. Es ist geplant, dass diese Studie nach Erhalt eines Ethikvotums an der Charité auch an mehreren Neurologischen Kliniken in Deutschland durchgeführt wird, sofern dies für andere Kliniken interessant ist. Dieses Vorgehen hätte den Vorteil, dass eine Abhängigkeit der gewonnenen Erkenntnisse vom Klinikstandort untersucht werden könnte.  Das primäre Ziel der Studie liegt daher in der Beantwortung der Frage, inwieweit es den Schlaganfallpatienten möglich ist, die im Rahmen eines standardisierten Aufklärungsgespräches zur Thrombolyse in der Notfallsituation vermittelten Informationen im verzögerten Abruf (60-90 Minuten nach der Aufklärung) zu rekapitulieren (Gruppe 1). Als weitere Zielgruppe sollen Angehörige (1. oder 2. Grades) der Schlaganfallpatienten in die Studie eingeschlossen werden, welche die Betroffenen ins Krankenhaus begleitet und ebenso am Aufklärungsgespräch zur Thrombolyse teilgenommen haben (Gruppe 2).  Darüber hinaus soll der Einschluss stationär behandelter Patienten mit einem ischämischen Schlaganfall erfolgen, die aufgrund bestehender Kontraindikationen (beispielsweise einer Erstvorstellung >4,5 Stunden nach  Symptombeginn) keine Thrombolyse erhalten haben. Diese sollen nach |

|  | Studieneinschluss eine Aufklärung über eine Thrombolyse erhalten (ohne diese appliziert zu bekommen) und im Anschluss befragt werden (Gruppe 3). Weiterhin sollen stationäre Patienten der Medizinischen Klinik für Kardiologie am Campus Benjamin Franklin, die ein vergleichbares kardiovaskuläres Risikoprofil aufweisen, nach Studieneinschluss in gleicher Weise eine Aufklärung über eine Thrombolyse erhalten (ohne diese appliziert zu bekommen) und im Anschluss befragt werden (Gruppe 4).  Neben dem Vergleich der ebene genannten vier Gruppen soll zudem geprüft werden, ob Patienten mit akutem ischämischem Schlaganfall die im Rahmen des standardisierten Aufklärungs-gespräches zur Thrombolyse vermittelten Informationen bei einem Abruf nach 24 Stunden in ähnlicher Weise rekapitulieren können (Gruppe 5), wie dies in der Gruppe der Schlaganfallpatienten mit einem Abruf nach 60-90 Minuten der Fall war (Gruppe 1).  Zudem soll bei Schlaganfallpatienten untersucht werden, ob die Fähigkeit zur Rekapitulation der im Rahmen des standardisierten Aufklärungsgespräches zur Thrombolyse vermittelten Informationen von der Schulbildung oder von der klinischen Bedeutung der Informationen abhängig sind. Dabei werden bestimmte klinische Informationen (bspw. Nutzen und mögliche Nebenwirkungen einer Thrombolyse) als bedeutsamer eingestuft, als andere Informationen (bspw. Applikationsart der Thrombolyse), die ebenfalls im Rahmen des standardisierten Aufklärungsgespräches zur Thrombolyse vermittelt werden.  Abschließend soll durch eine Beteiligung anderer Neurologischer Kliniken in Deutschland gezeigt werden, dass die Fähigkeit die im Rahmen des standardisierten Aufklärungsgespräches zur Thrombolyse vermittelten  Informationen bei Schlaganfallpatienten unabhängig vom Klinikstandort ist. |
| --- | --- |
| … Nutzen-Risiko-Abwägung | Die behandelnden Ärzte der Klinik für Neurologie werden vor Beginn der Studie über die Inhalte informiert. Zudem wird eine Liste mit den standardisierten Items der Aufklärung an die behandelnden Ärzte und Ärztinnen der Klinik für Neurologie ausgegeben, welche als Leitfaden für das Aufklärungsgespräch dienen soll. Hierdurch werden die Behandler für die vollständige Durchführung der Aufklärung sensibilisiert und die Qualität der Aufklärung nach ethischen Grundlagen gewährleistet. Für den Patienten bedeutet dies den Erhalt relevanter Informationen zum Krankheitsbild sowie zu dem möglichen Nutzen und den möglichen Risiken der Thrombolyse, selbst wenn die Therapie in Gruppe 2-4 nicht durchgeführt wird. Durch die erfolgende Wissensvermittlung könnten Patienten als auch Angehörige (Gruppe 2-4) im Falle eines späteren Schlaganfalls von diesen Informationen profitieren.  Nach der Analyse der im Rahmen der Studie erhobenen Daten könnten sich Erkenntnisse ergeben, die bspw. in einer methodischen Intervention zur Verbesserung der Aufklärungsprozedur münden könnten, von denen zukünftige Schlaganfallpatienten profitieren könnten.  Für Schlaganfallpatienten ist mit einer Studienteilnahme kein Risiko verbunden. Allerdings könnte in der Akutsituation die für die Studie notwendige Befragung durch den Studienarzt von dem Patienten (und/oder den Angehörigen) als belastend empfunden werden. Die Initiierung der Thrombolyse und die diesbezügliche Indikationsstellung werden durch eine im Nachgang mögliche  Studienteilnahme nicht beeinflusst. |
| **5. Studienziele** |  |
| …Primäre und sekundäre Ziele  …Hypothesen | Primäre Hypothese:  (1) Nach einem Aufklärungsgespräch zur intravenösen Thrombolyse bei akutem ischämischem Schlaganfall werden von den einwilligungsfähigen Schlaganfallpatienten (Gruppe 1) im Mittel 50% der genannten Fakten erinnert, wenn eine Abfrage 60 bis 90 Minuten nach erfolgter Thrombolyse-Aufklärung erfolgt.  Sekundäre Hypothesen:  (1) Nach einem Aufklärungsgespräch zur intravenösen Thrombolyse bei akutem |

|  | ischämischem Schlaganfall werden von den Angehörigen (1. oder 2. Grades) der Schlaganfallpatienten (Gruppe 2) im Mittel 75% der genannten Fakten erinnert, wenn eine Abfrage 60 bis 90 Minuten nach erfolgter Thrombolyse-Aufklärung eines Angehörigen erfolgt.   1. Einwilligungsfähige Patienten mit subakutem ischämischem Schlaganfall, die keine vorherige Thrombolyse erhalten haben (Gruppe 3), erinnern nach einem Aufklärungsgespräch zur intravenösen Thrombolyse im Mittel 75% der genannten Fakten, wenn eine Abfrage 60 bis 90 Minuten nach erfolgter Thrombolyse-Aufklärung (ohne nachfolgende Therapie) erfolgt. 2. Einwilligungsfähige Patienten ohne bisherigen Schlaganfall (Gruppe 4) erinnern nach einem Aufklärungsgespräch zur intravenösen Thrombolyse im Mittel 75% der genannten Fakten, wenn eine Abfrage 60 bis 90 Minuten nach erfolgter Thrombolyse-Aufklärung erfolgt. 3. Nach einem Aufklärungsgespräch zur intravenösen Thrombolyse bei akutem ischämischem Schlaganfall werden von den einwilligungsfähigen Schlaganfallpatienten im Mittel 50% der genannten Fakten erinnert, unabhängig davon, ob eine Abfrage etwa 24 Stunden (Gruppe 5) oder 60 bis 90 Minuten (Gruppe 1) nach erfolgter Thrombolyse-Aufklärung erfolgt. 4. Die Fähigkeit die im Rahmen eines Aufklärungsgespräches zur intravenösen Thrombolyse bei akutem ischämischem Schlaganfall benannten Fakten im Rahmen eines verzögerten Abrufes zu rekapitulieren ist von der Schulbildung, der Infarktlokalisation und dem Krankenhaus in dem die Aufklärung stattfindet unabhängig. 5. Die Fähigkeit die im Rahmen eines Aufklärungsgespräches zur intravenösen Thrombolyse bei akutem ischämischem Schlaganfall benannten Fakten im Rahmen eines verzögerten Abrufes zu rekapitulieren ist von der klinischen Wertigkeit der benannten Faktoren unabhängig. 6. Die Fähigkeit die im Rahmen eines Aufklärungsgespräches zur intravenösen Thrombolyse benannten Fakten im Rahmen eines verzögerten Abrufes zu   rekapitulieren ist vom Klinikstandort unabhängig. |
| --- | --- |
| **6. Studiendesign** | Prospektive multizentrische Beobachtungsstudie.  Die Studiendauer für die einzelnen Teilnehmer beträgt lediglich wenige Stunden nach Einholung der Einverständniserklärung.  Die gesamte Studiendauer richtet sich daher ausschließlich nach der Dauer der  Rekrutierung. |
| **7. Studienpopulation** |  |
| …Rekrutierungswege | Eingeschlossen werden stationär behandelte Patienten in den teilnehmenden Institutionen. |
| ...Einschlusskriterien | Einschlusskriterien für Schlaganfallpatienten, die eine Thrombolyse erhalten (Gruppe 1):   - Laufende oder unmittelbar beendete Thrombolyse aufgrund eines akuten ischämischen Schlaganfalls - Erstmalige Thrombolyse - Aufklärungsfähigkeit des Patienten/der Patientin - Alter ≥18 Jahre - Ausreichende Deutschkenntnisse - Befragung ist 60-90 min nach Beginn der Thrombolyse möglich - In diese Gruppe können auch Patienten mit akutem ischämischen Schlaganfall eingeschlossen werden, die eine Thrombolyse prinzipiell hätten erhalten können, diese jedoch in der Akutsituation abgelehnt haben.   Einschlusskriterien für Angehörige von Schlaganfallpatienten, die eine Thrombolyse erhalten (Gruppe 2): |

|  | - Laufende oder unmittelbar beendete Thrombolyse aufgrund eines akuten ischämischen Schlaganfalls bei einem Verwandten ersten oder zweiten Grades - Keine vorherige Thrombolyse gemäß Anamnese - Aufklärungsfähigkeit des Patienten - Alter ≥18 Jahre - Ausreichende Deutschkenntnisse   Einschlusskriterien für Schlaganfallpatienten, die keine Thrombolyse erhalten (Gruppe 3):   - Keine Thrombolyse gemäß Anamnese - Derzeitiger stationärer Aufenthalt aufgrund eines akuten ischämischen Schlaganfalls - Aufklärungsfähigkeit des Patienten/der Patientin - Alter ≥18 Jahre - Ausreichende Deutschkenntnisse   Einschlusskriterien für stationär behandelte Patienten, die keinen Schlaganfall haben (Gruppe 4):   - Keine Thrombolyse gemäß Anamnese - Derzeitiger stationärer Aufenthalt an der Charité - Aufklärungsfähigkeit des Patienten/der Patientin - Alter ≥18 Jahre - Ausreichende Deutschkenntnisse   Einschlusskriterien für Schlaganfallpatienten, die eine Thrombolyse erhalten, jedoch erst am Folgetag befragt werden (Gruppe 5):   - Laufende oder unmittelbar beendete Thrombolyse aufgrund eines akuten ischämischen Schlaganfalls - Erstmalige Thrombolyse - Aufklärungsfähigkeit des Patienten/der Patientin - Alter ≥18 Jahre - Ausreichende Deutschkenntnisse - Befragung ist 24 Stunden nach Beginn der Thrombolyse möglich |
| --- | --- |
| ...Ausschlusskriterien | Es wurden in Ergänzung zu den oben genannten Einschlusskriterien keine  additiven Ausschlusskriterien definiert. |
| **8. Individueller Studienablauf** |  |
| …Aufklärung und Einwilligung  …Allokation | Die Studienteilnehmer sollen schriftlich oder mündlich im Beisein eines Zeugen in die Teilnahme einwilligen (Einwilligungserklärung siehe Anlage). Die Einwilligung muss vor Beginn der Befragung vorliegen. Die Einwilligung durch einen gesetzlichen Vertreter ist nicht vorgesehen, da die Fähigkeit zur selbstständigen Einwilligung als Einschlusskriterium herangezogen wird.  Wie im Klinikablauf üblich, erfolgt die Anamneseerhebung und Erstuntersuchung der Schlaganfallpatienten entsprechend der aktuell bestehenden Standards der Schlaganfallbehandlung durch die behandelnden Fach- bzw. Assistenzärzte der Klinik für Neurologie. Besteht der Verdacht auf einen akuten Schlaganfall wird noch vor Durchführung der zerebralen Bildgebung, die eine Differenzierung zwischen einer zerebralen Ischämie und einer intrakraniellen Blutung ermöglicht, regelhaft ein so genannter “Stroke- Alarm“ ausgelöst, wodurch auch die Studienärzte der Klinik informiert werden. Der Studienarzt/die Studienärztin wird im Rahmen der geplanten Studie dem vom behandelndem Arzt der Klinik für Neurologie geführten Aufklärungsgespräch mit dem Schlaganfallpatienten (und seinen Angehörigen sofern vorhanden) über die Diagnose Schlaganfall und eine Thrombolyse beiwohnen. Das Aufklärungsgespräch entspricht den aktuellen Behandlungsstandards der Klinik für Neurologie. Die Entscheidung der Einwilligungsfähigkeit des Patienten obliegt hierbei dem behandelnden Arzt. Dieser erläutert dem Patienten (und seinen ihn begleitenden Angehörigen) die Erkrankung und die bestehende Behandlungsmöglichkeit einer intravenösen  Thrombolyse sowie die damit verbundenen Risiken. Zur Sichererstellung der |

|  | Vollständigkeit der Aufklärung erhält der behandelnde Arzt eine „Checkliste“ mit den durch ihn zu erwähnenden Items.  Der Studienarzt wird hierbei initial nicht aktiv am Gespräch teilnehmen, sondern lediglich eine beobachtende Rolle einnehmen und die Aufklärungsinhalte (vgl. Items des Aufklärungsgespräches) dokumentieren. Im Anschluss an das Gespräch soll auch im Sinne einer internen Qualitätssicherung, ein Feedback durch den Studienarzt an den aufklärenden/behandelnden Arzt erfolgen. Sollten wider Erwarten bestimmte Items des Studien-spezifischen Fragebogens bisher nicht angesprochen worden sein, werden diese angesprochen, um einen bestmöglichen Standard des Aufklärungsgespräches sicher zu stellen.  60-90 Minuten nach Beginn der Thrombolyse, die nach initialer Bolusgabe über eine Stunde intravenös gegeben wird, wird der Patient und sofern verfügbar auch die beim Aufklärungsgespräch ebenfalls anwesenden Angehörigen durch den Studienarzt kontaktiert. Es erfolgt dann die Information des Patienten (Gruppe 1) bzw. seiner Angehörigen (Gruppe 2) über die geplante Studie. Im Anschluss wird die Einwilligung zur Studienteilnahme eingeholt. Ist der Patient bzw. seine Angehörigen zu einer Studienteilnahme bereit, erfolgt das verzögerte Abfragen der Items des Aufklärungsgespräches.  Zudem ist der Einschluss von Patienten mit akutem ischämischem Schlaganfall geplant, welche aufgrund einer Überschreitung des Zeitfensters oder aus anderen Gründen bei Aufnahme keine Thrombolyse erhalten konnten (Gruppe 3). Diese Patienten werden nach kurzer Information über die geplante Studie und selbstständiger Einwilligung in die Studienteilnahme durch einen Arzt der Klinik für Neurologie über eine Thrombolyse aufgeklärt (ohne jedoch eine entsprechende Therapie erhalten zu haben) und ebenfalls 60-90 Minuten darauffolgend zu dieser Aufklärung befragt (ohne dass eine Thrombolyse erfolgt ist).  Darüber hinaus ist der Einschluss von Patienten mit ähnlichem kardiovaskulärem Risikoprofil geplant, welche im Rahmen eines stationären Aufenthaltes in der Medizinischen Klinik für Kardiologie am Campus Benjamin Franklin eine elektive Intervention erhalten, beispielsweise eine Kardioversion aufgrund eines Vorhofflimmerns (Gruppe 4). Diese Patienten werden durch einen Studienarzt während ihres stationären Aufenthaltes aufgesucht, über die geplante Studie informiert und um ihre Teilnahme gebeten. Nach selbstständiger Einwilligung werden sie durch den Studienarzt über eine Thrombolyse bei akutem ischämischen Schlaganfall aufgeklärt (ohne eine Behandlung zu erhalten). Das weitere Procedere erfolgt parallel zur oben beschriebenen Patientengruppe, d.h. es erfolgt eine erneute Visite durch den Studienarzt mit entsprechend um 60 bis 90-minütig verzögertem Abruf der Aufklärungs-Inhalte.  Als weitere Zielgruppe sollen zudem Patienten mit akutem ischämischen Schlaganfall dienen, welche etwa 24 Stunden (und nicht 60-90) Minuten nach Beginn der Thrombolyse in die Studie eingeschlossen und im Anschluss erstmals zu den Inhalten des Aufklärungsgespräches befragt werden (Gruppe 5).  Die Behandlung und medizinische Betreuung der Patienten wird während des gesamten Aufenthaltes durch die behandelnden Ärzte der aufnehmenden Klinik  gewährleistet. |
| --- | --- |
| **9. Unerwünschte Ereignisse** | Nicht zutreffend, da keine Erfassung von klinischen Endpunkten erfolgt. |
| **10. Biometrische Aspekte** |  |
|  | Ausgehend von der Hypothese, dass die Patienten im Mittel 50% der zuvor genannten Fakten wiedergeben können und dass die Standardabweichung zum Faktenwissen in der Patientengruppe 30% beträgt, lässt sich mit 100 Patienten ein 95% Konfidenzintervall für das mittlere prozentuale Faktenwissen mit einer Genauigkeit von ±6% ermitteln (44-56%). Bei einer kleineren / größeren Standardabweichung von 20% oder 40% wäre die Präzision des 95%Konfidenzintervalls entsprechend größer bzw. kleiner (±4% / ±8%). Für die  sekundären Hypothesen und anderen Gruppen werden ebenfalls Mittelwerte |

|  | zum erinnerten Faktenwissen aus dem Aufklärungsgespräch und 95%  Konfidenzintervalle berechnet. |
| --- | --- |
| **11. Datenmanagement** |  |
|  | Ein Abbruch der Studienteilnahme durch den Patienten selbst ist jeder Zeit und ohne die Nennung von Gründen möglich. Darüber hinaus liegt ein möglicher Abbruch der Studie, d.h. der Befragung, im Ermessen des Studienarztes und ist dabei abhängig von der individuellen Situation.  Mögliche Gründe für einen Studienabbruch wären eine Progression der bestehenden neurologischen Defizite oder sonstiger Komplikationen einer Thrombolyse.  Es sollen insgesamt 180 Schlaganfallpatienten (Gruppe 1: n=100, Gruppe 3: n=40; Gruppe 5: n=40) sowie 40-Angehörige (Gruppe 2) von Schlaganfallpatienten und 40 Patienten mit einem vergleichbaren kardiovaskulären Risikoprofil (Gruppe 4) eingeschlossen werden.  Die Datenerfassung erfolgt schriftlich auf dafür vorgesehenen CRF-Bögen. Diese werden in einem Ordner am Studienzentrum gesammelt und später wie gesetzlich vorgeschrieben archiviert. Die Probandenidentifizierung erfolgt für sämtliche die Studie betreffende Erhebungen in pseudonymisierter Form.  Die Datenerhebung und -verarbeitung der hier vorliegenden Studie erfolgen unter Beachtung des Datenschutzgesetzes. Eine Weitergabe von Daten oder  Ergebnissen an Dritte ist nicht vorgesehen. |
| **12. Ethische und rechtliche Aspekte** |  |
| …Richtlinien | Anwendung finden die Deklaration von Helsinki und die Maßgaben der Good Clinical Practice. |
| …Einreichung zur Ethikkommission | Erstvotum erfolgte im November 2016 durch die Ethikkommission der Charité – Universitätsmedizin Berlin.  Ethikvoten der für die einzelnen Studienzentren zuständigen  Ethikkommissionen wurden vor Beginn der lokalen Rekrutierung eingeholt. |
| …Datenschutz | Ein entsprechendes Votum der Charité – Universitätsmedizin Berlin liegt seit dem 11.11.2016 vor. Anwendung findet das Landeskrankenhausgesetz Berlin  (LKG) §25 Absatz 1 (Datenschutz bei Forschungsvorhaben). |
| …Versicherung | Nicht zutreffend. |
| **13. Anlagen** | Keine. |
