# Supplementary figures and images for: Ability of patients with acute ischemic stroke to recall given information on intravenous thrombolysis: Results of a prospective multicenter study
Source: Eur Stroke J. 2023 Jan 6;8(1):241–50. doi: 10.1177/23969873221143856 (PMC10069168; doi:10.1177/23969873221143856)

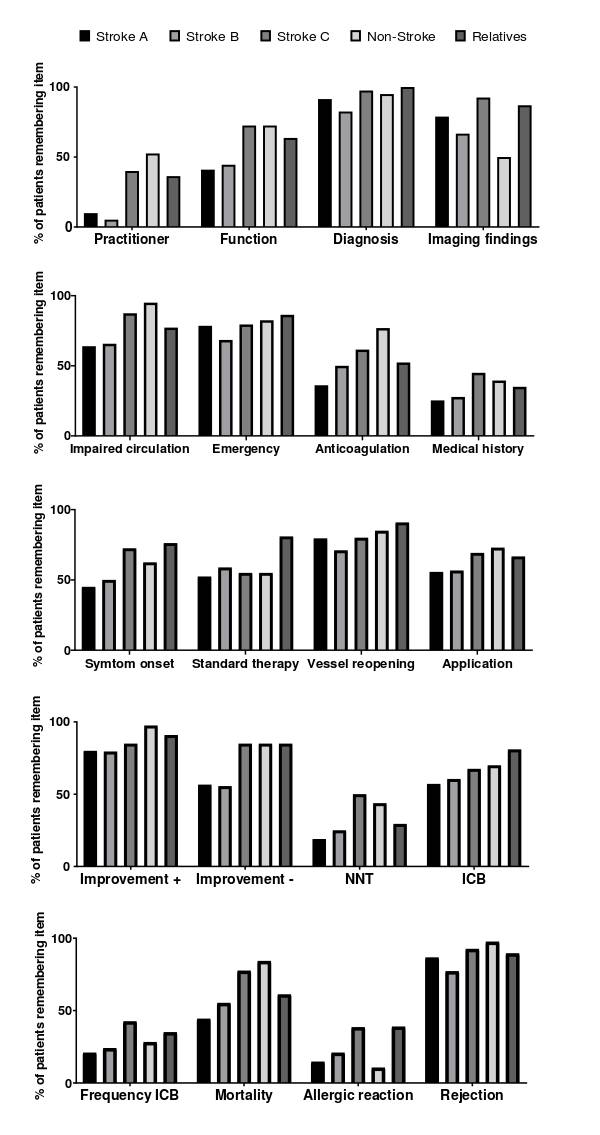

Supplement: sj-jpeg-4-eso-10.1177_23969873221143856 – Supplemental material for Ability of patients with acute ischemic stroke to recall given information on intravenous thrombolysis: Results of a prospective multicenter study [file sj-jpeg-4-eso-10.1177_23969873221143856.jpeg]

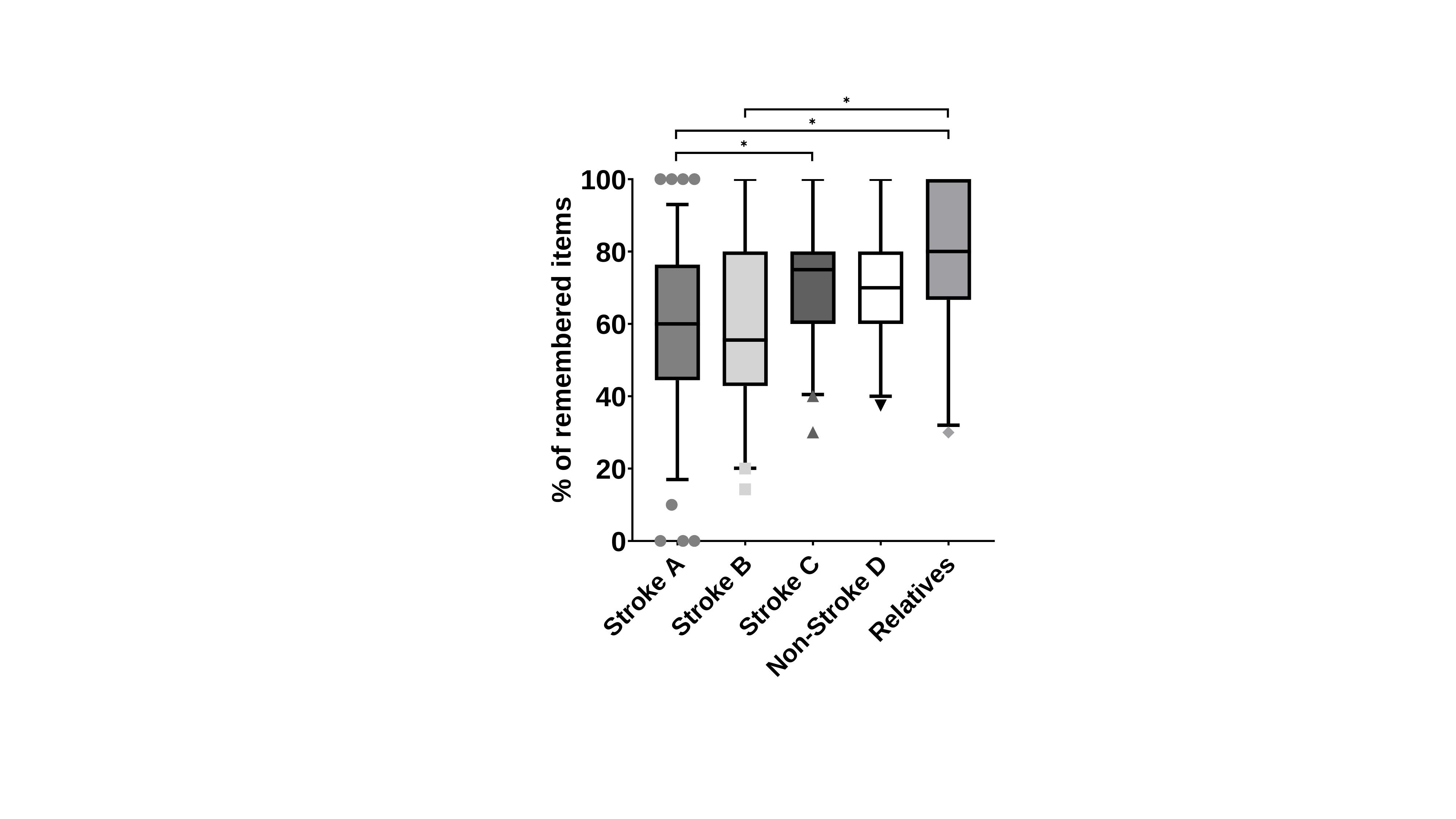

Supplement: sj-jpg-5-eso-10.1177_23969873221143856 – Supplemental material for Ability of patients with acute ischemic stroke to recall given information on intravenous thrombolysis: Results of a prospective multicenter study [file sj-jpg-5-eso-10.1177_23969873221143856.jpg]
