# Supplementary material for: Ability of patients with acute ischemic stroke to recall given information on intravenous thrombolysis: Results of a prospective multicenter study
Source: Eur Stroke J. 2023 Jan 6;8(1):241–50. doi: 10.1177/23969873221143856 (PMC10069168; doi:10.1177/23969873221143856)
Supplement: sj-pptx-3-eso-10.1177_23969873221143856 – Supplemental material for Ability of patients with acute ischemic stroke to recall given information on intravenous thrombolysis: Results of a prospective multicenter study [file sj-pptx-3-eso-10.1177_23969873221143856.pptx]

## Slide 1
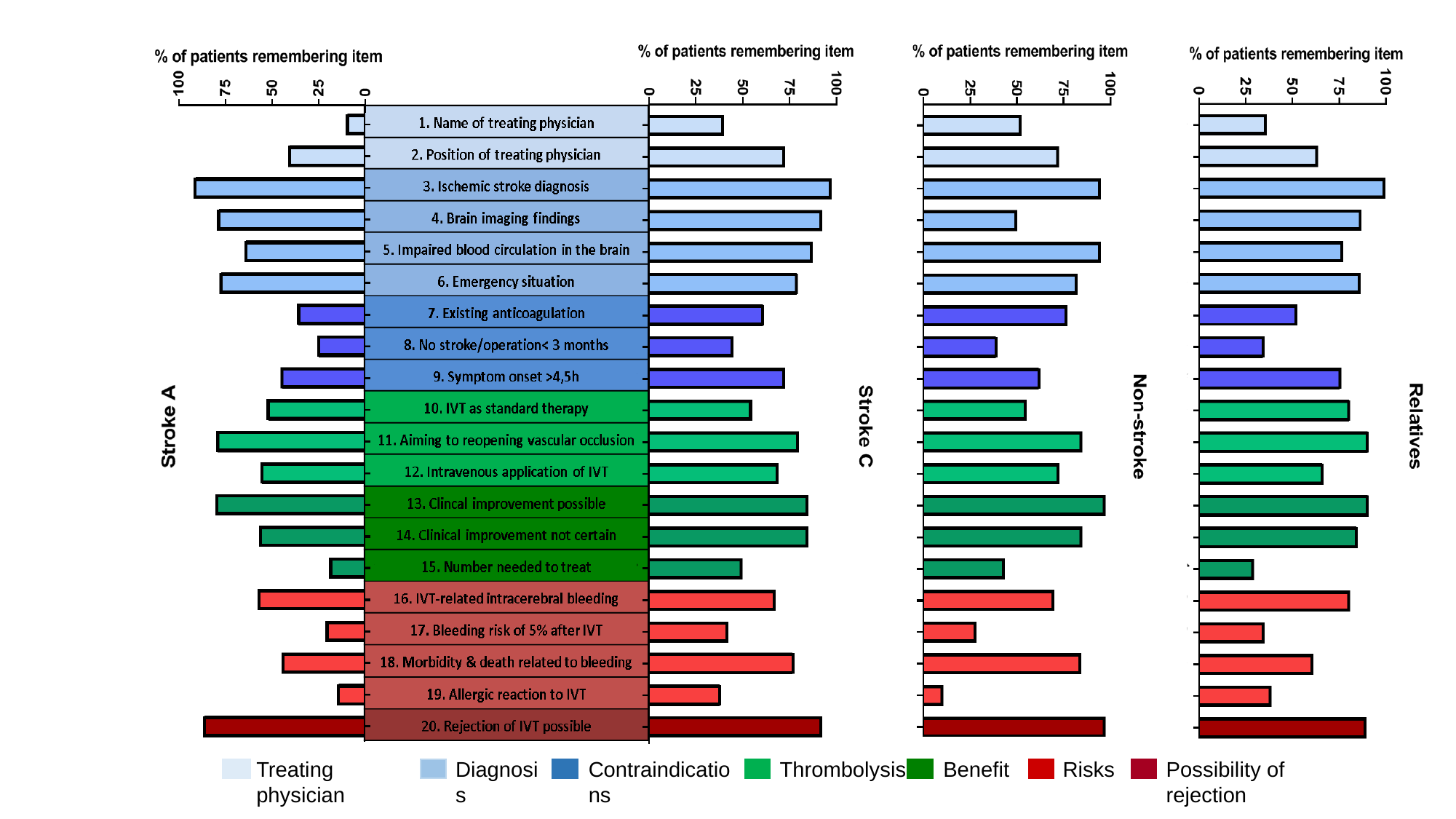

Treating physician
Diagnosis
Contraindications
Thrombolysis
Benefit
Risks
Possibility of rejection
